# Novel Molecular Insights into Leukemic Evolution of Myeloproliferative Neoplasms: A Single Cell Perspective

**DOI:** 10.3390/ijms232315256

**Published:** 2022-12-03

**Authors:** Sebastiano Rontauroli, Chiara Carretta, Sandra Parenti, Matteo Bertesi, Rossella Manfredini

**Affiliations:** 1 Centre for Regenerative Medicine “Stefano Ferrari”, Department of Biomedical, Metabolic and Neural Sciences, University of Modena and Reggio Emilia, 41125 Modena, Italy; 2 Centre for Regenerative Medicine “Stefano Ferrari”, Life Sciences Department, University of Modena and Reggio Emilia, 41125 Modena, Italy

**Keywords:** molecular pathogenesis, molecular landscape, single cell genomics, single cell transcriptomics myeloproliferative neoplasms, leukemic transformation

## Abstract

Myeloproliferative neoplasms (MPNs) are clonal disorders originated by the serial acquisition of somatic mutations in hematopoietic stem/progenitor cells. The major clinical entities are represented by polycythemia vera (PV), essential thrombocythemia (ET), and primary myelofibrosis (PMF), that are caused by driver mutations affecting *JAK2*, *MPL* or *CALR*. Disease progression is related to molecular and clonal evolution. PV and ET can progress to secondary myelofibrosis (sMF) but can also evolve to secondary acute myeloid leukemia (sAML). PMF is associated with the highest frequency of leukemic transformation, which represents the main cause of death. sAML is associated with a dismal prognosis and clinical features that differ from those of de novo AML. The molecular landscape distinguishes sAML from de novo AML, since the most frequent hits involve *TP53*, epigenetic regulators, spliceosome modulators or signal transduction genes. Single cell genomic studies provide novel and accurate information about clonal architecture and mutation acquisition order, allowing the reconstruction of clonal dynamics and molecular events that accompany leukemic transformation. In this review, we examine our current understanding of the genomic heterogeneity in MPNs and how it affects disease progression and leukemic transformation. We focus on molecular events elicited by somatic mutations acquisition and discuss the emerging findings coming from single cell studies.

## 1. Introduction

Myeloproliferative Neoplasms (MPNs) are clonal disorders originated by the acquisition of somatic mutations in hematopoietic stem cells (HSCs). Clinical entities belonging to this category are subdivided based on the presence/absence of the cytogenetic marker t (9; 22) in Chronic Myeloid Leukemia (CML) and Philadelphia-negative MPNs. The latter comprises, among the others, Essential Thrombocythemia (ET), Polycythemia Vera (PV) and Primary Myelofibrosis [1,2]. These disorders are all characterized by myeloproliferation, leading to the excessive production of terminally differentiated myeloid cells but involving different lineages: erythrocytosis is the hallmark of PV, while ET and PMF primarily affect megakaryocytes [3]. PMF is characterized by the development of bone marrow fibrosis due to the deposition of reticulin and collagen fibers, as a result of the altered interaction between stromal and hematopoietic cells belonging to the neoplastic clone [4,5]. Based on the magnitude of the lesion, pre-fibrotic (pre-PMF) and overt fibrotic (overt PMF) stages can be identified [1]. 

**Figure 1:**
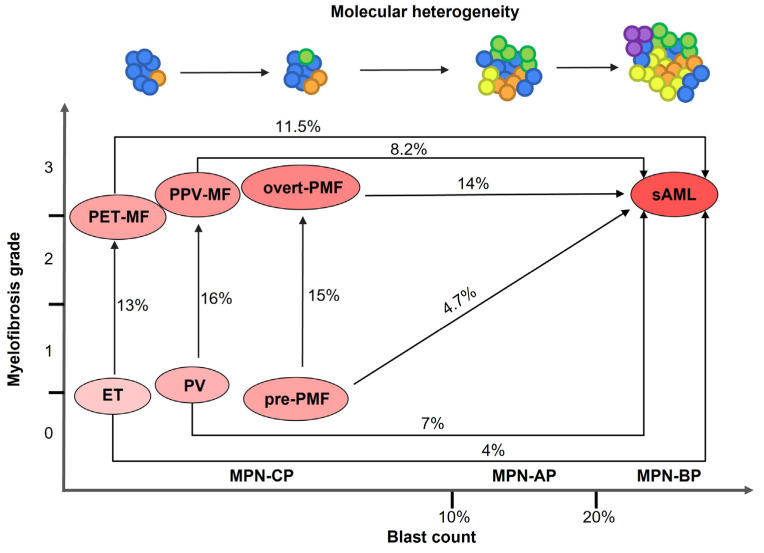
Routes of disease progression and evolution in MPNs. Owing to overlapping clinical features, different disorders belonging to the MPN family can evolve into one another. State transitions can be defined based on the degree of bone marrow fibrosis and/or the frequency of blasts in peripheral blood and bone marrow. Disease evolution is associated with increasing molecular heterogeneity. Percentages represent the observed frequency of disease progression and evolution in MPN patients. Shades of red represents disease aggressiveness. ET: Essential Thrombocythemia; PV: Polycythemia Vera; pre-PMF: pre-fibrotic Primary Myelofibrosis; PET-MF: post-ET Myelofibrosis; PPV-MF: post-PV Myelofibrosis; sAML: secondary Acute Myeloid Leukemia; MPN: Myeloprolifertive Neoplasm; MPN-CP: MPN chronic phase; MPN-AP: MPN accelerated phase; MPN-BP: MPN blast phase.

MPNs are closely related disorders displaying overlapping clinical and molecular features; patients may experience disease worsening and progression. In particular, ET and PV can develop bone marrow fibrosis leading to the diagnosis of secondary myelofibrosis (sMF, post-ET and post-PV myelofibrosis, respectively) [6] (Figure 1). MPN patients are at increased risk of thrombotic events and bleeding, however evolution into secondary Acute Myeloid Leukemia (sAML), also termed MPN blast phase (MPN-BP), represents the main causes of death [4]. MPN-BP is preceded by an accelerated phase (MPN-AP) and a chronic phase (MPN-CP) [7]. Post-MPN sAML has a particularly aggressive phenotype and displays inferior survival compared to MPN-CP, de novo AML and AML secondary to Myelodysplastic Syndromes (MDS) [8]. sAML does not respond to conventional therapies, and the only therapeutic option for patients is represented by allogeneic stem cell transplantation, which can provide only a short-term survival advantage [9] (Figure 1).

Leukemic transformation is more frequent in PMF (14%) compared to PV (7%) and ET (4%) [10]. Several clinical and laboratory parameters were identified as risk factors for leukemic transformation that may differ according to MPN diagnosis [11]. Interestingly, gene expression profiling of granulocytes from PMF and sMF patients highlighted the prognostic relevance of gene expression, leading to the description of a deregulated molecular signature that represent a risk factor for both inferior survival and leukemic transformation. Likewise, high levels of circulating long non-coding RNA LINC01268 was recently demonstrated to be correlated with inferior leukemia-free survival in MF [12,13]. Next generation sequencing (NGS) application to clinical practice allowed a better characterization of the genomic landscape of MPN-CP and BP, and led to the identification of molecular determinants of disease prognosis and leukemic transformation. 

In this review, we examine our current understanding of the genomic heterogeneity in MPNs and how it affects disease progression and leukemic transformation. We focus on molecular events elicited by somatic mutations acquisition, and discuss the emerging findings coming from single cell studies.

## 2. Molecular Pathogenesis of MPN-BP

Overlapping clinical manifestations and disease features among MPNs derive from their shared molecular landscape. MPN pathogenesis is promoted by mutually exclusive somatic “driver” mutations affecting three genes Janus kinase 2 (*JAK2*), MPL Proto-Oncogene or Thrombopoietin Receptor (*MPL*) and Calreticulin (*CALR*). The most frequent “driver” mutations are JAK2V617F, MPLW515L and CALRdel52 or CALRins5, all converging towards the constitutive activation of the JAK/STAT signaling pathway. Besides its role in regulating megakaryocyte differentiation, it was demonstrated that mutated *CALR* compromises the ability of hematopoietic cells to respond to oxidative stress, thus leading to genomic instability and accumulating DNA damage [14,15,16]. The molecular mechanisms triggered by these mutations to confer a selective growth advantage to hematopoietic stem/progenitor cells (HSPCs) have been extensively described and discussed by other authors [17,18,19]. 

**Figure 2:**
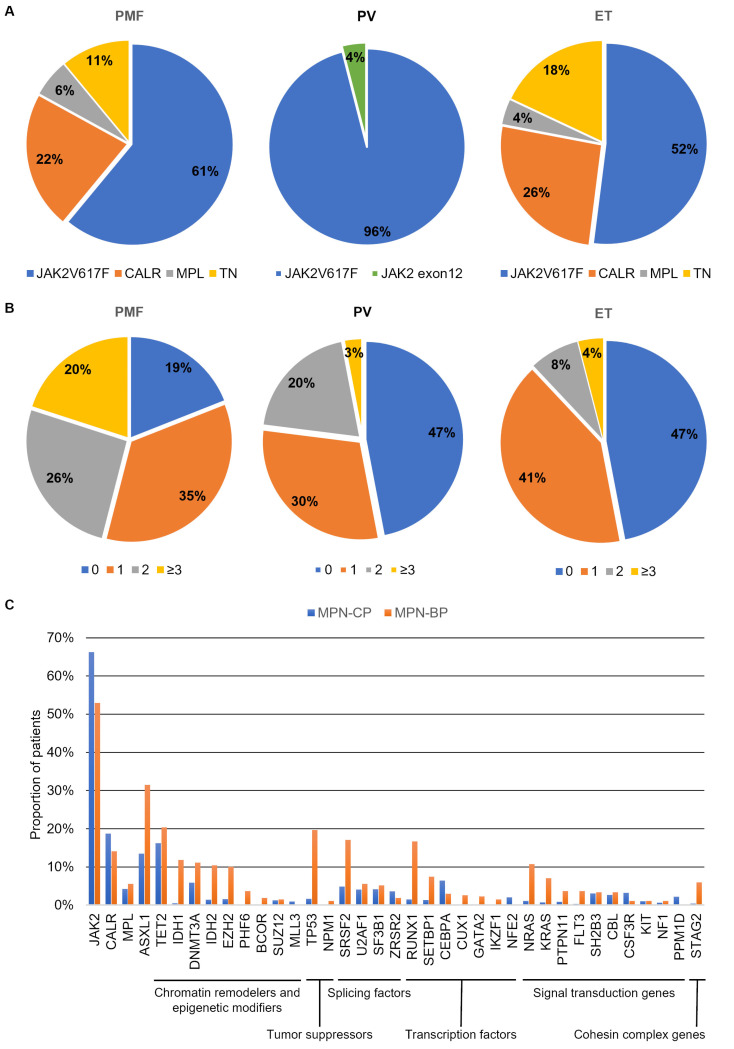
Molecular landscape in MPN-CP and MPN-BP. Panel (**A**) reports the frequency of patients harboring one of the three driver mutations considering PMF, PV and ET separately. In Panel (**B**) the proportion of patients harboring 0, 1, 2 or more than or equal to 3 variants other than the driver mutations is represented. Histogram in Panel (**C**) compares the frequency of patients harboring each mutation in MPN-CP and MPN-BP. Data adapted from: Tefferi et al., 2016 [21], Tefferi et al., 2016 [22], Grinfeld et al., 2018 [24], Venton et al., 2018 [25], Lasho et al., 2018 [26] and McNamara et al., 2018 [27].

The frequency of each driver mutation changes according to diagnosis. Most PV patients harbor the canonical JAK2V617F mutation (96%), while the remaining 4% display *JAK2* variants affecting exon 12. Conversely, the JAK2V617F mutation has been identified in only 55% and 60% of ET and PMF patients, respectively [4,20]. On the other hand, *MPL* and *CALR* mutations are identified only in ET and PMF. Almost 3% of ET and 5% of PMF cases harbor an *MPL* variant, whereas *CALR* mutations’ frequency is about 25–30% in both ET and PMF (Figure 2A) [21,22]. A substantial proportion of patients affected by ET or PMF (10–15%) do not harbor any of the three driver mutations, and are therefore termed triple negative (TN) (Figure 2A). TN patients display the worst prognosis and inferior leukemia-free survival [10].

The observation that the three clinical entities are associated with the presence of the same driver mutations reinforces the notion that disease manifestation and characteristics are influenced by other concurrent factors. Among them, the molecular landscape of MPNs plays a central role, since patients can be affected by additional somatic mutations and cytogenetic aberrations that cooperate with the “driver” events to define disease aggressiveness, response to therapy, progression and evolution to sAML [23]. In particular, PMF is associated with increased molecular complexity compared to both ET and PV, as a higher proportion of patients harbor additional genetic variants and display a higher number of concurrent gene mutations and chromosomal changes [21,22,24] (Figure 2B). Grinfeld and colleagues developed a genomic classification of MPNs, including 8 disease subgroups defined by the presence of specific variants; each group was characterized by a diverse propensity to progress towards myelofibrosis or to evolve to MPN-BP. In particular, pathogenic variants affecting epigenetic modifiers, RAS pathway genes, splicing factors and Tumor Protein 53 (*TP53*) identified the subgroups with the highest risk of transformation to sAML [24]. Compared to MPN-CP, sAML patients were more likely to harbor variants in chromatin remodelers and epigenetic modifiers, *TP53*, Serine and Arginine Rich Splicing Factor 2 *(SRSF2*), transcription factors (i.e., *RUNX1*, *CUX1*, *GATA2*, *IKZF1*) and signal transduction genes (i.e., *NRAS*, *KRAS*, *PTPN11*, *FLT3*) (Figure 2C).

### 2.1. Chromatin Remodelers and Epigenetic Modifiers

Additional Sex Combs Like 1 (*ASXL1*) is the second most frequently mutated gene in PMF (36%) [21] and encodes a member of the polycomb repressive complex (PRC). *ASXL1* is mainly affected by heterozygous frameshift or nonsense mutations in the C-terminal Plant Homeodomain (PHD) finger responsible for the interaction with PRC1 and PRC2 proteins [28]. Another component of the PRC2, Enhancer of Zeste 2 Polycomb Repressive Complex 2 (*EZH2*), stands among the most frequently mutated genes in MPN-BP (Figure 2C). *EZH2* encodes a histone methyltransferase responsible for the tri-methylation of H3K27 residue that promotes heterochromatin formation and gene silencing [29]. Most mutations affecting *EZH2* are loss of function, and result in increased chromatin relaxation and higher transcriptional activity of specific loci such as Homeobox A9 (*HOXA9*) [30,31]. The same effect is induced by *ASXL1* variants; therefore, these mutations affect HSPCs self-renewal and differentiation and are involved in MPN pathogenesis (Figure 2). 

**Figure 3:**
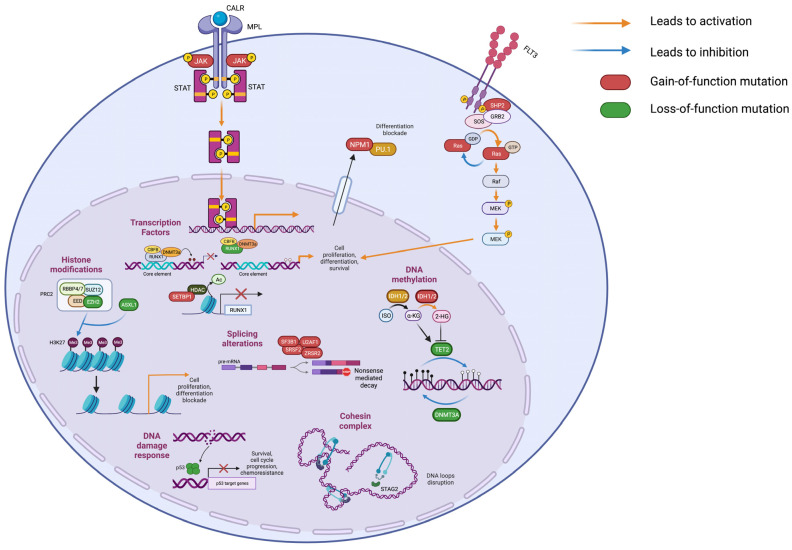
Molecular events responsible to MPN onset and evolution. This image summarizes the molecular processes and pathways involved in MPN onset and leukemic evolution described in the main text. Abbreviations: JAK2: Janus Kinase 2; CALR: Calreticulin; TPO-R: Thrombopoietin Receptor; STAT: Signal Transducer and Transcription Activator; PRC2: Polycomb Repressive Complex 2; ASXL1: Additional Sex Combs Like 1; EZH2: Enhancer of Zeste Homolog 2; EED: Polycomb protein EED; SUZ12: Polycomb protein SUZ12; RBBP4/7: Histone-binding protein RBBP4/7; Ac: Acetylation; Me3: three-methylation; H3K27: Histone 3 Lysin 27; DNMT3A: DNA methyltransferase 3 α; TET2: Ten-Eleven-Translocation 2; IDH1/2: Isocitrate Dehydrogenases 1/2; ISO: Isocitrato; α-KG: α-ketoglutarate; 2-HG: 2-Hidroxyglutarate; p53: Tumor Protein 53; SRSF2: Serine/Arginine Rich Splicing Factor 2; SF3B1: Splicing factor 3B subunit 1; U2AF1: Splicing factor U2AF 35 kDa subunit; U2AF1: U2 small nuclear ribonucleoprotein auxiliary factor 35 kDa subunit-related protein 2; RUNX1: Runt-related transcription factor 1; SETBP1: SET binding protein 1; FLT3: FMS-like tyrosine kinase 3; SHP2: SH2 containing protein tyrosine phosphatase-2; Ras: Rat sarcoma virus; Raf: RAF proto-oncogene serine/threonine-protein kinase; MEK: Mitogen-activated protein kinase kinase; ERK: extracellular signal-regulated kinase; STAG2: Stromal Antigen 2; NPM1: Nucleophosmin; PU.1: Transcription factor PU.1. Created with BioRender.com.

Besides histone modification, DNA methylation plays a crucial role in regulating gene expression. Genes involved in this process are frequently mutated in both CP and BP MPNs. Among them, DNA Methyltransferase 3 Alpha (*DNMT3A*) is frequently mutated in both MPN-CP and MPN-BP (Figure 2C). *DNMT3A* mutations result in impaired methylating activity, leading to altered chromatin accessibility [32]. In MPN mouse models, *DNMT3A* loss led to the decreased expression of differentiation factors, upregulation of multipotency genes and inflammatory pathways and myelofibrotic progression [33]. On the contrary, Tet Methylcytosine Dioxygenase 2 (*TET2*) encodes a methylcytosine dioxygenase involved in DNA demethylation. Loss-of-function mutations in TET2 catalytic domain led to DNA hypermethylation (Figure 3). In vivo, *TET2* impairment cooperates with JAK2V617F, leading to the expansion of the HSC compartment and the development of an aggressive myeloproliferative phenotype [34,35]. Isocitrate Dehydrogenases (IDH) are NADP-dependent enzymes responsible for oxidative decarboxylation of isocitrate to α-ketoglutarate in the Krebs cycle [36]. Among the family members, *IDH1* and *IDH2* are affected by loss-of-function mutations in MPNs. Missense mutations in *IDH1/2* genes reduce their affinity for isocitrate and confer the ability to convert α-ketoglutarate to 2-hydroxyglutarate, which can bind to TET2 and inhibit its demethylating activity [37]. Furthermore, it has been demonstrated that 2-hydroxyglutarate prevents histone demethylation, leading to a differentiation block [38] (Figure 3). The combined expression of a JAK2V617F and *IDH1/2* mutant in mice induce a MPN phenotype characterized by impaired differentiation of HSPCs with accumulation of increased immature progenitors [39].

### 2.2. Tumor Suppressor Genes

*TP53* encodes a tumor suppressor that responds to DNA damage by inducing specific transcriptional programs for DNA repair, cell cycle arrest and apoptosis. *TP53* is generally affected by missense mutations that may cause either loss of tumor suppression effect, novel gain-of-functions, or have a dominant negative effect (Figure 3). A recent study by Bernard et al. highlighted the importance of TP53 allelic state in MDS, as only multi-hit *TP53* mutations were predictive of inferior survival and increased risk of leukemic transformation, whereas monoallelic mutations did not alter the outcome and response to therapy [40]. In vivo studies support the pivotal role of *TP53* in MPN leukemic transformation, since it was demonstrated that it is essential for controlling HSCs quiescence and its absence induces cell cycle entry [41]. Moreover, Rampal and colleagues demonstrated that *TP53* deletion cooperates with JAK2V617F expression in mice to induce AML development [42].

### 2.3. Splicing Factors

*SRSF2* is a spliceosome component that recognizes exon splicing enhancers (ESE) on primary transcripts. The most frequent genetic alterations in MPNs are missense variants involving the hotspot Proline 95 on exon 2 (p.P95H and p.P95L) that impair protein function by affecting *SRSF2* preferred RNA recognition sequence, thus leading to mis-splicing events (Figure 3). Mis-splicing of *EZH2* induces a decrease in its expression due to nonsense-mediated decay that impairs HSC differentiation [43]. Moreover, mutated *SRSF2* influences gene transcription by inducing the preferential expression of the short RUNX1a (Runt-related transcription factor 1) isoform, which has been reported to be overexpressed in AML [44,45] and promotes the expansion of murine HSCs [46].

### 2.4. Transcription Factors

*RUNX1* is a transcription factor that binds DNA and dimerizes with the core binding factor (CBF-β) through its runt homology domain (RHD). RUNX1 regulates normal hematopoiesis and influences HSC differentiation by recruiting DNMT3A to its target genes inducing their repression [47]. This transcription factor is frequently mutated in MPN-BP (Figure 2.C) and is affected by missense, frameshift and nonsense variants that cause the loss of its function and induce a dominant-negative effect [48]. The ectopic expression of RUNX1 p.D171N in CD34+ cells from MPN-CP patients enhanced its self-renewal capacity, and induced proliferation of primitive progenitors and immature myeloid cells [49]. *RUNX1* is essential in leukemic transformation induced by SET binding protein 1 (*SETBP1*) mutations. *SETBP1* missense variants are frequent in MPN-BP and stabilize SETBP1 protein by decreasing its degradation. In a mouse model, overexpression of *SETBP1* in hematopoietic progenitors resulted in the development of myeloid leukemia characterized by reduced expression of *RUNX1* due to deacetylation of histones in the gene promoter mediated by Histone deacetylase 1 (HDAC1) [50].

### 2.5. Signal Transduction Genes

FMS-like tyrosine kinase 3 (FLT3) is a tyrosine-kinase receptor expressed by HSPCs and involved in regulation of proliferation, cell survival and differentiation. Upon interaction with its ligand, FLT3 activates phosphatidylinositol-3-kinase (PI3K), RAS and signal transducer and activator transcription factor (STAT) cascades [51]. *FLT3* most frequent pathogenic variants include internal tandem duplications (FLT3-ITD) of the juxtamembrane domain that abolish its autoinhibitory function, and missense mutations in the tyrosine-kinase domain (FLT3-TKD), both responsible for FLT3 constitutive activation [51]. 

*RAS* genes encode small GTPases that represent key elements in mitogen-activated protein kinase (MAPK) signaling pathways. The most frequently mutated members in MPNs are NRAS Proto-Oncogene, GTPase (*NRAS)* and KRAS Proto-Oncogene, GTPase (*KRAS)* which display heterozygous missense variants, NRAS p.G12D and KRAS p.G12D being the most frequent. These variants activate growth signals by reducing intrinsic GTP hydrolysis and promoting resistance to GTPase-activating proteins (GAPs) [52]. 

Furthermore, the tyrosine phosphatase Protein tyrosine phosphatase, nonreceptor type 11 (PTPN11) is frequently mutated in MPN-BP. This protein is responsible for RAS dephosphorylation, and mutations observed in AML increase PTPN11 activity, leading to the activation of the MAPK pathway [53] (Figure 3).

Signaling gene mutations are strongly associated with leukemic transformation in MPN. *FLT3*, *NRAS*, *KRAS* and *PTPN11* variants have been detected in a small fraction of MPN and MDS/MPN cases and are considered late events in MPN disease progression towards sAML [54]. In mice, knock-in of FLT3-ITD or FLT3-TKD, as well as NRAS p.G12D and KRAS p.G12D, induced a myeloproliferative neoplasm, and an AML phenotype was obtained by combination with other variants [55].

### 2.6. Cohesin Complex Genes

Stromal Antigen 2 (*STAG2*) is a member of the cohesin complex [56,57]. *STAG2* is affected by nonsense and frameshift mutations or splice site changes that lead to protein truncation or exon skipping [58] (Figure 3). In mice, *STAG2* deletion in hematopoietic stem and progenitor cells induces myeloid dysplasia, and is responsible for increased self-renewal and impaired differentiation [59]. In AML, *STAG2* mutations are frequently accompanied by *RUNX1* variants [60]. The combined loss of *STAG2* and *RUNX1* induces a MDS phenotype in vivo by deregulating gene expression through disruption of chromatin loops, leading to the expansion of myeloid progenitor cells [61].

## 3. Clonal Phylogenesis in MPNs

In MPNs, disease phenotype and leukemic transformation are influenced not only by the presence of a complex genotype and co-occurring somatic variants, but also by a mutation acquisition order and combination of variants within neoplastic cells. These aspects influence neoplastic cell functions and corresponding disease manifestations. As recently demonstrated by several authors, driver mutation acquisition by HSPCs can precede MPN clinical manifestation by many years. Reconstruction of time of mutation acquisition, based on whole genome sequencing of colonies, revealed that JAK2V617F or CALR mutations can appear in a founding HSC decades before MPN diagnosis, even in utero, demonstrating that mutated clone fitness and expansion are influenced by other factors like concurrent somatic alterations [62,63,64].

Genotyping of colonies from patients harboring one driver and at least one additional variant allowed for the reconstruction of the mutation acquisition order in clonal hematopoietic cells [65]. Using this approach, it was recently demonstrated that *ASXL1* and *EZH2* mutations are usually acquired as first mutational events, and are therefore responsible for the establishment of clonal hematopoiesis [66]. On the other hand, *TET2* and *DNMT3A* mutations can both precede or follow the acquisition of JAK2V617F mutation. When JAK2V617F is the first acquired variant (JAK2-first), patients display increased frequency of circulating megakaryocyte/erythroid progenitors (MEPs) differently from patients with *TET2* as the first mutated gene (TET2-first), who display increased common myeloid progenitors (CMP). In TET2-first patients, single mutant cells dominate HSPC compartment while most erythroid colonies also display the *JAK2* mutation, suggesting that *TET2* mutations sustain clonal expansion, whereas *JAK2* variant is responsible for increased proliferation and expansion of the MEP compartment [67]. Moreover, Nangalia and colleagues demonstrated that when the *DNMT3A* mutation is acquired as first, single mutant colonies are detectable. Whereas, when it is preceded by *JAK2* or *MPL* driver mutations, the double mutant clone dominates the hematopoietic compartment. This demonstrated that *JAK2*/*MPL* single mutated clones have a competitive disadvantage compared with *DNMT3A* single mutant or double mutant clones. The mutation acquisition order influences the disease phenotype, since DNMT3A-first patients were affected by ET, while JAK2-first patients were more likely to be affected by PV or MF [68]. 

Genomic analysis of single colonies was also used to describe events associated with leukemic transformation. Evidence of diverse patterns of disease evolution came from the observation that in many cases, JAK2V617F positive MPN-CP may give rise to JAK2 WT MPN-BP [69,70]. Genomic studies in different hematopoietic cell populations from both CP and BP or genotyping of colonies demonstrated that two routes for leukemic transformation are most likely to occur: (1) CP and BP cells arise from a common founder clone that precedes the acquisition of JAK2V617F mutation responsible for CP disease, or (2) the two clones responsible for CP and BP arise from two independent ancestors that acquired different somatic mutations [69,71]. These observations were confirmed and expanded in recent years, taking advantage of the most recent single cell approaches [72]. Compared with single cell genomics, genotyping of colonies does not necessary reflect the real subclones’ frequency, because some of them might display a growth advantage in vitro that leads to biased mutation detection compared to variant allele frequency (VAF) derived from bulk analysis [62,63,73].

## 4. Single Cell Technologies

In recent years, single cell technologies have been developed to obtain a comprehensive picture of normal and pathological cellular phenomena. Bulk analysis does not allow a full understanding and reconstruction of cellular or clonal diversity that resides in genetically heterogeneous systems such as neoplasms. On the other hand, single cell analysis enables dissecting the biological asset of mixed populations at different omics levels. These technologies can retrieve information from different cellular macromolecules: DNA, identifying Single Nucleotide Variants (SNVs), Copy Number Variations (CNVs) and chromatin accessibility status; RNA, both nuclear and total mRNA; and proteins. Moreover, multi-omics studies can be performed thanks to the combination of two or more aforementioned approaches. 

Single cell genomics has been widely used to describe the molecular heterogeneity within neoplastic cell populations, since it is able to detect specific mutations within each cell. One of the first approaches implemented to perform this analysis consists of single cell isolation, followed by Whole Genome Amplification (WGA) [74]. This method, like colony genotyping, can be employed for a few hundred cells. To overcome these limitations, microfluidic approaches such as Tapestri Platform by Mission Bio have been developed to perform automated genomic analysis of thousands of single cells. Targeted panels can be designed to detect SNVs and small indels, also determining their zygosity in each cell. VAF inferred from single cell data is consistent with the one originated from bulk NGS [73,75]. Unlike prior methods, single cell genomic analysis is able to detect SNVs down to 0.1% VAF. For this reason, single cell analysis is a robust method to detect rare genetic alterations in MPN-CP, which may eventually lead to leukemic transformation. Nevertheless, the main limitation of this technique actually relates to the use of pre-designed panels, including only a limited number of genomic regions frequently mutated in MPNs and AML.

Single cell RNAseq (scRNA-seq) is the most employed single cell technology, enabling the identification of cell types within a heterogeneous biological sample and describing the transcriptomic changes induced by drug treatments and/or disease progression in clusters of neoplastic cells. Moreover, scRNA-seq can be a tool for reconstructing cell developmental pathways branching from stem cells to the last differentiation stages. Currently, two main experimental workflows are used to perform this assay: SMART-seq [76] and 10× Genomics technology [77]. SMART-seq represents a low-throughput technique which allows to perform whole transcriptome analysis on a limited number of cells, while the 10x instrument prioritizes the number of analyzed cells without being able to detect full-length transcripts.

The development of single cell omics analysis has paved the way for the evaluation of cell type-specific regulation by means of multi-omic analysis [78,79]. Among the methods designed to perform integrative analysis of genomic and transcriptomic data are TARGET-seq [80,81], genome and transcriptome sequencing (G&T-seq) [82] and Genotyping of Transcriptomes (GoT) [83]. The GoT-splice method was also recently designed to monitor alternative splicing events through long-read sequencing [84].

Different methods, including Cellular Indexing of Transcriptomes and Epitopes by sequencing (CITE-seq), have been developed to evaluate the immunophenotypic asset of a cell together with its gene expression [85]. Recently, an integrated analysis of surface immunophenotypic markers and a targeted DNA mutational landscape was made available by Mission Bio for Tapestri platform, applying the same principles employed in CITE-seq to single cell genomics. All the aforementioned methods require a high number of input cells, as approximately only 10% of them are retrieved after processing.

As of today, the clinical application of single cell techniques is limited by the high costs required by instruments needed both to generate and sequence single-cell omics libraries. Moreover, highly qualified personnel both in wet-lab work and bioinformatic data analysis is necessary to obtain reliable results.

## 5. Single Cell Genomic Studies in MPNs

Given the clonal nature of hematological malignancies, single cell technologies are powerful tools in deciphering single cell molecular heterogeneity to obtain a comprehensive description of neoplastic onset and progression. In particular, new insights on MPN progression have resulted from single cell genomic analysis [86,87].

Zygosity assessment has proved to be greatly important in the understanding of molecular events elicited during MPN pathogenesis and leukemic transformation. Traditionally, splicing factor mutations have always been considered to occur in heterozygosity, as their allele frequency in bulk populations rarely exceeds 50%. Nevertheless, the single cell genomic analysis of a PMF patient that harbored a *SRSF2* mutation, with a bulk allele frequency of approximately 50%, highlighted the presence of WT, heterozygous and homozygous cells for this variant [73]. Analogous results were obtained by Meira and colleagues, who analyzed two patients displaying similar JAK2V617F VAF from bulk sequencing. At the single cell level, the same VAF could be generated by the presence of a large clone harboring the heterozygous mutations, or a small clone homozygous for the JAK2V617F variant [80]. Therefore, the bulk evaluation of allele frequency did not consider the zygosity of the subclones generated from this variant.

Single cell analysis is also an important tool for detecting genetic alterations, leading to leukemic transformation in a very small cell fraction of MPN-CP samples that cannot be detected by bulk NGS [88]. This was the case of a PMF patient we studied in a recently published work; by means of single cell genomic analysis of CD34+ cells, we were able to identify a small clone harboring FLT3-TKD mutation in the chronic phase of the disease, that was not revealed by bulk NGS in the same patient. The expansion of this clone was responsible for the leukemic transformation observed in this patient; therefore, its precocious detection might have been relevant for clinical management [73]. In line with our observations, Guess and colleagues confirmed the high resolution of single cell genomics by defining the molecular events associated with leukemic evolution in a restricted cohort of MDS patients. In three out of eighteen studied patients, leukemic transformation was associated with the expansion of a hematopoietic clone whose molecular profile was identified in only a few cells in the disease’s chronic phase [75]. 

As a whole, single cell analysis enables the reconstruction of the mutational acquisition order, the clonal composition of the neoplastic population and the definition of clonal dynamics associated with leukemic transformation.

### 5.1. Mutation Acquisition Order

Several studies have highlighted how the chronological acquisition order of somatic mutations during neoplastic pathogenesis is crucial for determining clonal fate and disease progression. Results of single cell genomic analyses of different myeloid malignancies including MPN, MDS and AML, highlighted underlying genetic similarities. Pathogenic variants affecting the epigenetic remodelers *DNMT3A*, *TET2*, *ASXL1* and *IDH1/2* (collectively defined DTAI mutations) are among the most frequently detected variants, and were often identified as the initiating mutational event [88]. This observation implies that DTAI mutations are often responsible for the onset of clonal hematopoiesis (CH), as suggested by the observation that they are among the most frequent events in elderly healthy individuals displaying this condition [89,90]. However, this “rule” cannot be fully generalized because *TET2* can be acquired both as a first or secondary mutation, and DNMT3A p.R882H usually occurs as a secondary event and is frequently associated with leukemic transformation, at least in MDS patients [75,80,88].

On the other hand, mutations in signal transduction genes or *TP53* variants are highly frequent in MPN and MDS patients who undergo sAML evolution. Single cell genomics of paired samples from 18 MDS patients evolved to sAML have demonstrated that *TP53* mutations were detected in both the founder and the dominant clone. This was in line with bulk enhanced whole-genome sequencing, that was able to detect *TP53* variants in both MDS and AML samples [75,91]. Therefore, *TP53* mutation can represent an early mutational event in MDS patients. The same was observed in MPN by Lundberg and colleagues, who analyzed serial samples from patients who evolved to sAML using bulk NGS. In these patients, *TP53* mutations were detectable at low frequency years before leukemic transformation, which occurred upon the loss of the *TP53* WT allele [65]. Four routes for leukemic transformation guided by *TP53* have been recently described using TARGET-seq: (1) acquisition of biallelic *TP53* mutations, (2) acquisition of one *TP53* variant followed by deletion of WT allele, (3) concomitant evolution of 2 subclones with different TP53 mutations, and (4) expansion of a clone harboring *TP53* biallelic mutation while being negative for an MPN driver in a JAK2V617F-positive patient [92]. Single cell studies involving both MPN and MDS demonstrated that mutations affecting signaling genes are sub-clonal, are acquired in a later stage of the disease, and are usually detected in clone(s) responsible for leukemic transformation [73,88]. In MDS, these variants were frequently preceded by mutations on transcription factors [75,91].

Splicing factor and cohesin mutations are frequently associated with MPN-BP, and often occur as secondary events in clones already harboring chromatin remodelers’ variants [73,91]. In the context of MDS, Gaiti et al. applied single cell GoT-splice to highlight how *SF3B1* mutation occurs mainly in the MEP subpopulation [84].

### 5.2. Clonal Architecture

Single cell genomics discriminates between genetically different clones in a heterogeneous population as demonstrated by Miles and colleagues, who studied 146 samples from 123 patients including CH, MPNs and AML. They observed higher clonal complexity in AML samples compared with both CH and MPN. Indeed, AML displayed an increased number of mutations and clones, in line with results from other authors [40,73,88]. In particular, *FLT3* or *RAS*-mutated AML patients displayed higher clonal heterogeneity when compared to CH, MPN or AML driven by other genetic alterations [88]. Similarly, *TP53*-mutated sAML showed more variants than *TP53*-mutated MPN patients who do not undergo leukemic transformation [92]. Interestingly, MDS differs from MPNs since the transition to sAML is not defined by an increase in mutational events and/or clonal diversity [75].

Despite the high clonal diversity, AML patients display only a restricted number of dominant clones [88]. Moreover, it was observed that the mutational burden of a clone is not strictly connected with its fitness. Miles and colleagues observed that, in AML samples, the increased complexity did not correlate with an increased number of variants within the dominant clone, demonstrating that dominance is not necessarily linked to the amount of mutations carried by a clone. Indeed, specific mutations in *JAK2*, *IDH2* and/or *NPM1* might primarily contribute to fitness, and have been reported as prominently present in the dominant clone [88]. On the other hand, *FLT3* and *RAS* mutations were found in both minor and dominant subclones. 

**Figure 4:**
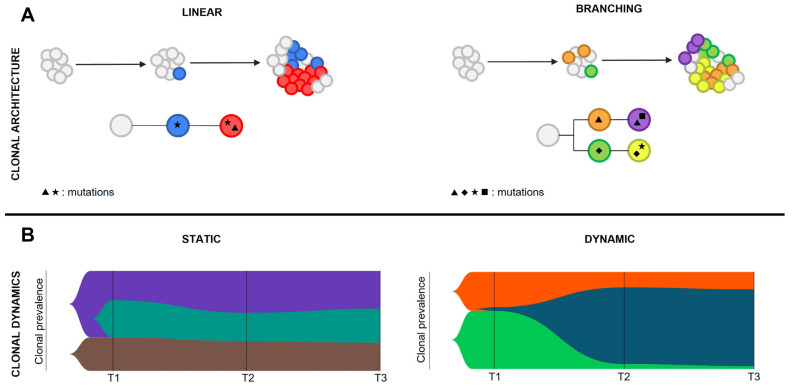
Clonal architecture and dynamics. Figure represents the possible clonal structures observed in MPN patients who evolved to sAML as reconstructed according to single cell genomics studies. Panel (**A**) shows the possible patterns of clonal architecture, while in pale (**B**) are reported fish plots representing the possible clonal dynamics.

Co-occurrence and mutual exclusivity patterns can be identified among mutations within clones, giving rise to peculiar genetic landscapes [93]. Clonal architecture is the static image of the neoplastic subclones at a given time, and is defined by their hierarchy and relative abundance. Two clonal architecture patterns have been described: linear, when mutations are acquired from a common mutated ancestor in a stepwise manner, or branching, when cells undergo parallel divergent evolution from the founder malignant clone(s) (Figure 4). Chromatin remodelers and epigenetic modifiers mutations do often co-occur within the dominant clone, suggesting their involvement in defining clone fitness. On the other hand, signaling gene variants are mutually exclusive in both MDS and MPN evolving to sAML. RAS pathway variants can be found in the same patient but seldom in the same clone, thus generating a branching clonal architecture [75,88,91]. The existence of branched clonal structures within neoplastic cell populations implies that clonal architecture cannot be predicted based on VAF coming from bulk analysis, but needs to be defined by using colony genotyping or more advanced single cell genomic techniques.

### 5.3. Clonal Dynamics

The single cell genomic approach is mainly employed to study complex clonal hierarchies and characterize rare genetic alterations that lead to resistance to pharmacological treatments and disease progression. In particular, clonal dynamics are defined by clonal evolution, the change of clonal architecture over time. The analysis of clonal evolution patterns is the key to understanding AML progression, since the dominant clone that guides leukemic transformation is usually very small or absent in the chronic phase of the disease. Clonal evolution can be static, when the clonal frequency does not change significantly over time and no clones get eradicated or dramatically expand; or dynamic, when disease progression is defined by marked shifts in clonal architecture (Figure 4). Epigenetic modifiers’ mutations are mostly involved in disease initiation rather than leukemic transformation. For this reason, they are mainly associated with a static evolution in the transition between MPN and sAML. In contrast, signaling mutations and *TP53* pathogenic variants are frequently associated with a dynamic evolution, in accordance with the pivotal role they play in triggering leukemic transformation [75,91]. As previously described, RAS pathway mutations mostly arise independently, thus generating different malignant clones with various degrees of fitness [75,88,91]. Multi-hit *TP53* mutations can occur either in the same clone in which the first *TP53* mutational event occurred, resulting in linear evolution, or give rise to a branching architecture [92]. In the MPN context, AML evolution may also be marked by the erasing or strong reduction of the clone harboring the driver mutation, suggesting that these mutations do not significantly contribute to leukemic transformation [92]. 

Dynamic evolution may also be elicited by a therapeutic treatment. For instance, FLT3-inhibitor treatment on AML patients can induce the eradication of the FLT3-mutated clone, paving the way for other therapy-resistant clones who will gain a proliferative advantage after the clonal sweeping [88].

## 6. Single Cell Transcriptomics and Proteomics

During the past decades, bulk transcriptomic analysis allowed the identification of molecular mechanisms that concur to the onset and evolution of MPNs, including the altered expression of non-coding RNAs [94,95]. 

It is now clear that neoplastic cells in MPN comprise numerous clones with diverse mutation signatures. The high genomic heterogeneity within the neoplastic cell population relates with differences in cell function and behavior. Single cell transcriptomics allows to disentangle this complexity by the detection of cell clusters distinguished according to their gene expression profile. This approach can describe molecular mechanisms responsible for disease onset and evolution that might be elicited by specific mutational events. ScRNA-seq of lin-CD34+ HSPCs from MF patients harboring JAK2V617F or *CALR* mutations and healthy donors (HDs) allowed the identification of clusters composed by stem cells and progenitors belonging to the various hematopoietic lineages. MF HSPCs displayed a megakaryocyte bias compared with cells from HDs, and a higher expression of megakaryocyte markers like Integrin Subunit Alpha 2b (*ITGA2B*, CD41) and Megakaryocyte and Platelet Inhibitory Receptor G6b (*MPIG6B*, G6B), the latter being able to distinguish between JAK2V617F mutated and unmutated cells within the same sample. ScRNA-seq highlighted altered transcriptional programs specific for different cell clusters. Compared with HD cells, MF megakaryocyte progenitors (MKP) displayed higher expression of fibrosis-related genes and transcriptional programs related to metabolic pathways (e.g., fatty acid metabolism and oxidative phosphorylation) and inflammation, such as tumor necrosis factor-α (TNF-α) and interferon signaling [96]. By means of TARGET-seq, Meira and colleagues could identify correlations between single cell gene expression signatures and genotype. They analyzed the transcriptional profile of HSPCs from 8 MF patients and 2 HDs, demonstrating that JAK2V617F positive cells, both homozygous or heterozygous, displayed enrichment in inflammation pathways like TNF-α, TGFβ and interferons, but also TP53, Wnt/β-Catenin, and hedgehog signaling. Inflammatory pathways were also enriched in unmutated cells from MF patients, supporting the pivotal role of the altered microenvironment in disease pathogenesis [80]. Single cell transcriptomics was recently employed to study the phenotypic heterogeneity of bone marrow (BM) stromal cell subpopulations, and describe their interaction with neoplastic HSPCs in both mouse models and MPN patients [97,98]. In PMF, mesenchymal stromal cells (MSC) displayed reduced expression of genes supporting HSCs and increased production of extracellular matrix proteins. In particular, the alarmin S100A8/S100A9 complex turned out as a potential antifibrotic target whose expression was increased in MSCs subpopulations expanded in PMF patients [98]. Likewise, AML cells induced a global reprogramming of BM MSCs, reducing osteogenic differentiation and the expression of factors supporting HSCs in their niche [97].

These results confirmed the contribution of inflammation to MPN onset. The same pathway turned out as a key mediator for MPN progression and evolution. In a recent work, Meira and colleagues performed TARGET-seq experiments to study disease evolution in patients harboring *TP53* mutations, an already known driver of MPN-BP. In these patients, leukemic transformation was triggered by loss of the WT allele, leading to expansion of a *TP53* multi-hit dominant clone. It was possible to compare TP53 mutant cells with WT ones. Two clusters of TP53 mutant cells were identified based on gene expression profile, one cluster with an erythroid bias, demonstrating the role of *TP53* impairment in altering HSPCs differentiation, the other one with leukemic stem cell (LSC) features, characterized by the expression of cell cycle and inflammation-related genes. On the other hand, TP53 WT cells from sAML differed from those derived from HDs or MF due to the impaired differentiation, expression of Wnt/β-Catenin genes and downregulation of cell cycle genes. *TP53* WT sAML cells were also characterized by the enrichment of gene signatures related to inflammatory mediators, such as TNFα, IFNγ, TGFβ and IL2, that may account for the phenotypic effect mediated by microenvironmental factors. By comparing heterozygous TP53 mutated cells from patients in MPN-CP before leukemic transformation with the same cells from patients who did not evolve to sAML, the central role of inflammation in leukemic evolution was highlighted. Indeed, the increased expression of interferon response genes, DNA repair and oxidative phosphorylation was observed. A transplant mouse model demonstrated that inflammation, triggered by poly(I:C) and mediated by IFNγ, sustained the expansion of *TP53* heterozygous cells [92].

In a recently published work, we studied the molecular mechanisms driving leukemic transformation in a patient with PMF who evolved to sAML while receiving Ruxolitinib. Genomic analysis revealed that leukemic transformation was sustained by the expansion of clones harboring *TP53* and *FLT3* mutations. This result was confirmed by single cell transcriptomics, revealing the increased expression of *FLT3* and enrichment in FLT3 signatures in BP cells. Trajectory analysis revealed that BP cells displayed a more primitive phenotype compared with CP cells, which were enriched in more differentiated cell states. Interestingly, our analysis highlighted an immunosuppressive role for Ruxolitinib treatment. IFN signaling, which is mediated by the JAK/STAT pathway, turned out to be inactivated in AP and BP samples, consistent with JAK2 inhibition. As a result, leukemic cells, particularly the progenitor compartment, displayed reduced expression of type I and II human leukocyte antigens (HLA) and β2 microglobulin (B2M) protein, which sustained the immune-escape of leukemic cells together with the activation of the programmed cell death (PD-1/PD-L1) axis. Impaired interferon signaling correlated also with reduced differentiation and apoptosis of leukemic cells [73]. As a whole, these studies demonstrated that single cell transcriptomics allows the identification of molecular mechanisms responsible for disease onset and evolution, that may be different from one patient to the others based on the underlying genetic heterogeneity.

## 7. Conclusions

Philadelphia-negative MPNs are a group of heterogeneous disorders sharing specific biological and clinical features. Myeloproliferation, the shared hallmark of these hematologic neoplasms, is sustained by the constitutive activation of the JAK/STAT signaling pathway downstream of hematopoietic growth factor receptors, primarily EPO-R and TPO-R. This effect is elicited by three driver mutations affecting *JAK2*, *MPL* and *CALR* that may also account for some of the differences observed. Nevertheless, most of the diversity among MPNs is explained by the underlying genetic variability. Besides driver mutations, patients may harbor many other somatic variants that influence disease manifestation, progression and outcome. Studies performed over the years focused primarily on the clinical and prognostic implications of the genetic underlying complexity of MPNs, allowing the identification of the molecular features associated with inferior survival and increased risk of disease evolution. Indeed, the main cause of death for MPN patients is represented by evolution to sAML, which is refractory to conventional therapy. To date, the only effective therapeutic approach is represented by allogeneic stem cell transplantation. Risk of sAML evolution relates to the underlying genetic complexity and it is increased in PMF, which displays higher intra-tumor heterogeneity. Bulk genomic studies demonstrated that, compared with PV and ET, PMF is associated with a higher number of concurrent genetic alterations that contribute to the more aggressive disease phenotype. 

The development of NGS technologies allowed the identification of an increasing number of genetic variants within the same sample, allowing to correlate disease evolution with specific mutations. The main drawback of this approach relies on the fact that it is performed in bulk, and therefore does not allow distinguishing between the different subclones that compose the neoplastic population. 

Single cell approaches provide an opportunity to disentangle this complexity. This is of primary importance to understand the underlying molecular mechanisms that account for disease onset and evolution. As demonstrated by the first single cell studies applied to MPN cases evolved to sAML the fitness of each subclone, therefore its contribution to disease pathogenesis and progression is strictly related to its molecular signature. Bulk sequencing approaches evaluating the VAF of each mutation cannot precisely reconstruct the clonal architecture of the neoplastic population, and fail to detect small cell clusters of prognostic relevance. Single cell genomic studies are able to precisely reconstruct mutation acquisition order and clonal evolution, allowing the identification of gene variants that might co-occur within the same cells and those that are mutually exclusive due to their redundant functions. Information provided by single cell genomic studies could be useful to clarify how the order of mutation acquisition impacts on different MPN phenotypic manifestations, while functional studies will be needed to reveal the molecular consequences of variant acquisition. Moreover, single cell studies can detect rare cell clusters that eventually expand and drive the leukemic transformation. Retrospective studies on small patient cohorts demonstrated that these clones can be detected early, years before the diagnosis of sAML, and provide the first evidence for the diagnostic and prognostic advantages given by single cell analysis. In the next few years, the extension of these results to larger patient cohorts will strengthen these observations, allowing to discriminate the real prognostic power of single cell genomics. The precocious identification of small subclones characterized by the presence of known detrimental variants will be helpful in terms of risk stratification. This will support clinical decision to address high risk patients for more effective precision medicine strategies or bone marrow transplantation. To date, their application in clinical practice is mainly limited by the high costs due to the need for cutting-edge instruments and highly qualified personnel, both in wet-lab and data analysis.

Single cell transcriptomics and genomics allow the description of molecular events associated with disease onset and evolution. Thanks to multi-omic approaches that combine different technologies to inspect multiple information layers in the same cell, it is now possible to overcome the limitations of bulk analysis and describe the precise molecular events elicited by specific gene mutations and combinations of variants in genetically or immunophenotypically defined cell populations. It has been made possible to describe the biological processes impaired due to the presence of specific gene mutations that directly contribute to increasing neoplastic cell fitness. When applied to the study of patients evolved to sAML, single cell studies allowed the identification of molecular mechanisms that may account for the biological features of the expanding clone, such as increased proliferation and immune escape, and reduced apoptosis and differentiation. These results pave the way for novel functional studies that are mandatory to validate the role of combination of variants in leukemic transformation. The first evidence suggesting a possible clone selection induced by therapeutic treatment were provided as well, highlighting the need to deepen this subject to discriminate the possible therapy drawbacks and have a better representation of pathways responsible for therapy resistance. 

The upcoming knowledge resulting from single cell studies will be pivotal for the dissection of molecular heterogeneity underlying MPN onset and progression. These techniques provide accurate and specific molecular information, whilst returning a comprehensive picture of the clonal cross-talk. Moreover, identification of novel biomarkers of disease progression will help to define patient-tailored therapeutic approaches that might contribute to a better management of MPN, thus reducing or preventing the risk of leukemic transformation.
